# Retraction

**DOI:** 10.1111/cas.15626

**Published:** 2022-11-13

Retraction: Hui Ren, Guoqin He, Zhiyuan Lu, Qianting He, Shuai Li, Zhexun Huang, Zheng Chen, Congyuan Cao, Anxun Wang, “Arecoline induces epithelial‐mesenchymal transformation and promotes metastasis of oral cancer by SAA1 expression”, Cancer Science 2021,112 (6), pp. 2173–2184.

The above article, published online on 24 February 2021 in Wiley Online Library (https://onlinelibrary.wiley.com/doi/full/10.1111/cas.14866), has been retracted by agreement between the authors, the journal Editor in Chief, Masanori Hatakeyama, and John Wiley and Sons Australia, Ltd.

The retraction has been agreed following an investigation based on allegations raised by a third party. Several duplicated cell micrographs were found in figures 1, 4, 5 and S4. The authors provided new images and stated that a substantial part of raw data was lost in the meantime. However, concerns about the integrity of the data remain for the newly provided figures. Accordingly, the conclusions of this article are considered invalid.

